# Iron and Folic Acid Supplementation in Pregnancy: Findings from the Baseline Assessment of a Maternal Nutrition Service Programme in Bangladesh

**DOI:** 10.3390/nu14153114

**Published:** 2022-07-28

**Authors:** Sk Masum Billah, Camille Raynes-Greenow, Nazia Binte Ali, Farhana Karim, Sharif Uddin Lotus, Rashidul Azad, Mayang Sari, Piyali Mustaphi, Md. Maniruzzaman, Shah Mohammad Mustafizur Rahman, Michael John Dibley, Patrick John Kelly, Shams El Arifeen

**Affiliations:** 1 Maternal and Child Health Division, International Centre for Diarrhoeal Disease Research, Bangladesh, Dhaka 1212, Bangladesh; nazia.ali@icddrb.org (N.B.A.); farhana.karim@icddrb.org (F.K.); lotus.sharif@icddrb.org (S.U.L.); rashidul.azad@icddrb.org (R.A.); shams@icddrb.org (S.E.A.); 2 Sydney School of Public Health, The University of Sydney, Sydney, NSW 2006, Australia; camille.raynes-greenow@sydney.edu.au (C.R.-G.); michael.dibley@sydney.edu.au (M.J.D.); p.kelly@sydney.edu.au (P.J.K.); 3 United Nations Children’s Fund, Dhaka 1207, Bangladesh; msari@unicef.org (M.S.); pmustaphi@unicef.org (P.M.); 4 National Nutrition Service, Institute of Public Health Nutrition, Directorate General of Health Services, Ministry of Health and Family Welfare, Dhaka 1212, Bangladesh; manirzdr@gmail.com (M.M.); mmm09us@gmail.com (S.M.M.R.)

**Keywords:** iron and folic acid, supplement, maternal anaemia, pregnancy, effective coverage, antenatal care, Bangladesh

## Abstract

Effective coverage of antenatal iron and folic acid (IFA) supplementation is important to prevent adverse maternal and newborn health outcomes. We interviewed 2572 women from two rural districts in Bangladesh who had a live birth in the preceding six months. We analysed the number of IFA tablets received and consumed during pregnancy and examined the factors influencing IFA consumption by multiple linear regression and user adherence-adjusted effective coverage of IFA (consuming ≥180 IFA tablets) by Poisson regression. Overall, about 80% of women consumed IFA supplements in any quantity. About 76% of women received antenatal care at least once, only 8% received ≥180 IFA tablets, and 6% had user adherence-adjusted coverage of antenatal IFA supplementation. Multivariable analysis showed a linear relationship between the number of antenatal care (ANC) visits and the number of IFA supplements consumed, which was modified by the timing of the first ANC visit. Women’s education, free IFA, and advice on IFA were also associated with higher IFA consumption. Interventions targeting at least eight ANC contacts, starting early in pregnancy, providing advice on the importance of IFA, and providing IFA supplements in higher quantity at ANC contacts are likely to increase effective coverage of antenatal IFA supplementation.

## 1. Introduction

Anaemia in pregnancy remains a global public health concern affecting nearly 35 million women and is the most prevalent in South Asia [1,2]. The 2020 global nutrition reported that all 194 countries are currently “off course” to achieve the 2025 target of a 50% reduction of anaemia in women of reproductive age [1]. Iron deficiency is the biggest contributor to anaemia in pregnancy, adversely affecting maternal, foetal, and newborn health and survival [3,4,5]. Iron deficiency anaemia in pregnancy also has detrimental longer-term outcomes on the child’s neurodevelopment and risk of intellectual disability [6]. The national estimate of pregnancy anaemia in Bangladesh suggests that nearly half of women were anaemic [7]. A recent study in Tangail, a rural district of Bangladesh, identified a 48% prevalence of anaemia in pregnant women [8]. According to the national estimate, the prevalence of anaemia in women was higher in rural areas (45%) than in urban areas (36%) [7].

Daily oral supplementation of a combination of iron and folic acid (IFA) is the most cost-effective intervention to reduce iron deficiency anaemia in pregnancy, prevent adverse maternal and perinatal outcomes, and improve infants’ linear growth [9,10]. WHO recommends a universal daily supplementation of IFA (30–60 mg elemental iron and 0.4 mg folic acid) for at least 180 days during pregnancy [11]. A 2020 update of the recommendations on antenatal care interventions included context-specific supplementation of multiple micronutrient supplements, including iron and folic acid [12]. The 2021 Lancet nutrition series also supports antenatal IFA and multiple micronutrient supplements as priority nutrition interventions during the “first 1000 days of life” [13,14].

In 2001, the government of Bangladesh adopted universal daily oral supplementation of IFA (60 mg elemental iron and 0.4 mg folic acid) for pregnant women, starting from pregnancy detection until birth. It has continued to reinforce IFA’s importance and improvement of coverage in national policy, strategy, and action plans [15,16,17,18]. The current National Nutrition Services Operation Plan of the fourth sector program further emphasised improving effective coverage of IFA supplementation (compliance to recommend ≥180 IFA tablets) during pregnancy [19]. Based on the latest recommendation by WHO, the National Nutrition Service has initiated a maternal nutrition demonstration programme to identify implementation strategies for multiple micronutrient supplements with 30 mg elemental iron and 0.4 mg folic acid through antenatal care services at public facilities [20]. In Bangladesh, pregnant women can receive IFA supplements (tablets) at antenatal care contacts at public health facilities, receive or purchase IFA during private ANC or home visits by community health workers, or purchase IFA from pharmacies over the counter [21,22]. Although widely available and relatively cheap, coverage and adherence to IFA supplementation in pregnancy have been poor [23,24]. The latest national estimate suggested ~74% crude coverage of antenatal IFA supplementation (consumed IFA of any quantity) but only 46% of women consumed ≥90 IFA tablets [25].

Effective coverage of antenatal IFA supplementation is essential to achieve its expected benefits on maternal and child health [26]. Recent frameworks for measuring the effective coverage of health and nutrition interventions provide a cascade-based framework, extending the traditional measurement of crude coverage to input, quality, user adherence, and outcome-adjusted coverage [26,27]. Effective coverage of antenatal IFA supplementation is usually measured by user adherence-adjusted coverage, e.g., adherence to the recommended ≥180 IFA supplements during pregnancy [26]. However, the national survey in Bangladesh did not report adherence to the recommended minimum dose (≥180 IFA tablets). Some earlier studies qualitatively explored IFA supplementation policy, programmes, and barriers to IFA supplementation [21,22,28]. However, very few studies reported the factors influencing the coverage of antenatal IFA supplementation based on population-level data [29]. Nonetheless, no study has reported the number and sources of IFA supplements that women receive during pregnancy, which is important for programmatic inputs on where to intervene to address the coverage gap of IFA. In the baseline situation assessment for the National Nutrition Service’s maternal nutrition demonstration programme, we explored three broad areas concerning antenatal IFA supplementation: (i) the source and consumption of IFA during pregnancy; (ii) faltering points for adherence to recommended IFA supplementation; (iii) factors influencing consumption of IFA and user adherence-adjusted effective coverage of IFA supplementation.

## 2. Materials and Methods

### 2.1. Setting and Study Design

We conducted the study in Kurigram and Bhola Districts, northern and southern districts of Bangladesh, respectively. Approximately four million people live in these two districts and have predominantly rural and agriculture-based livelihoods [30]. Both districts are below national averages in most maternal and child health service indicators [31].

The study is a randomised controlled trial to assess the effectiveness of a demonstration programme for improving coverage and quality of maternal nutrition services through the public health ANC platform. A detailed description of the trial design and interventions have been reported elsewhere [32]. In brief, in the cluster trial, we randomly assigned 40 unions (the lowest administrative units in Bangladesh) into intervention and control arms in a 1:1 allocation ratio. Each union’s primary health care facilities usually consist of one union health and family welfare centre and two to three community clinics serving approximately 25,000–30,000 residents. The demonstration programme aims to strengthen three priority nutrition interventions delivered through ANC contacts. The interventions were appropriate dietary counselling, gestational weight gain monitoring, and introducing MMS with iron and folic acid instead of the existing IFA supplements, based on WHO’s recent recommendation [12]. The programme emphasised improving health facility readiness to provide quality nutrition services at ANC by ensuing relevant logistics and equipment, improving health care provider’s knowledge and skills, ensuring a consistent supply of MMS, and strengthening monitoring and supervision. A set of community-based demand creation strategies aimed to increase the utilisation of primary health care centres for ANC services. The control clusters had the usual practice of maternal nutrition interventions. The programme implementation started in June 2022. As part of the baseline situation analysis for the programme, we conducted a household survey to explore the existing practice and coverage of the priority maternal nutrition interventions, including receiving and consuming IFA supplements during pregnancy.

### 2.2. Sampling and Data Collection

**Figure 1:**
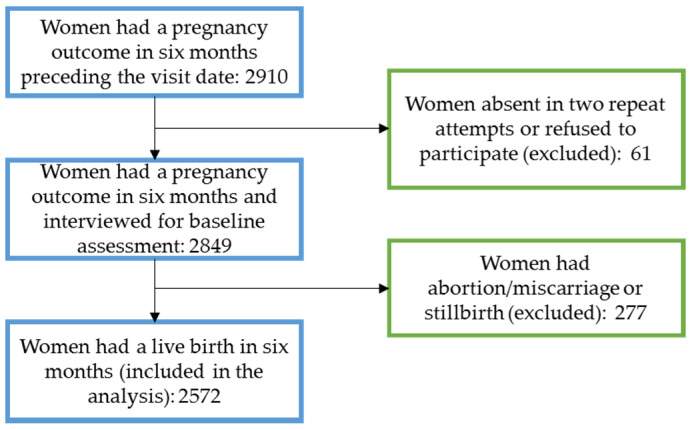
Participant flow diagram.

We interviewed women who had a pregnancy outcome within six months preceding the interview in the household survey. We selected the women by a multistage cluster sampling process. At first, in consultation with the National Nutrition Service, we purposively selected three subdistricts from each district where the demonstration programme will start in the first phase. Then, we selected 40 out of 60 available unions from the three subdistricts based on their similarity on a score created from population size, area, female literacy rate, sanitation coverage, availability of functioning primary health care facility, childhood immunisation coverage, and utilisation of public facilities for ANC and birth. We selected seven village clusters (sampling units) in the next stage, each of 250–300 households from each union following a probability proportional to size sampling approach. Finally, we identified and interviewed all (approximately ten on average) women who had a pregnancy outcome within six months in each selected cluster. Women who had a live birth outcome were included in the final analysis for this paper (Figure 1). Trained data collectors interviewed the selected women using a structured questionnaire developed based on the national demographic and health survey questionnaire [25]. We included questions on the number and source of IFA supplements received during pregnancy to the existing questions on the consumption of IFA supplements. We completed the survey between September and December 2020.

### 2.3. Outcome Variables

Outcome variables for this analysis were the total number of antenatal IFA supplements that women with a live birth outcome within six months before the interview had received and consumed. We asked the mothers to self-report the number of IFA tablets they received or purchased during pregnancy. We recorded the number of tablets they received or purchased during and outside the ANC contacts separately. Women also reported IFA sources in response to a multiple-answer question. Then, women were asked about the total number of IFA tablets they consumed during pregnancy. Following the effective coverage measurement cascade proposed by Marsh et al. [27] and Amouzou et al. [26], we created a binary outcome variable for user adherence-adjusted effective coverage of antenatal IFA supplementation if women consumed the recommended ≥180 IFA tablets during pregnancy (yes = 1, no = 0). We also conducted a modified falter point estimate of IFA supplementation using the method developed by Fiedler and colleagues [24], which identifies the critical falter points in receiving and consuming the recommended ≥180 IFA tablets. Our framework expands the falter point schematics proposed by Fiedler and colleagues and includes five sequential falter points [24]. The first falter point is women receiving ANC during pregnancy. The second falter point is women who received ANC or purchased IFA in any amount. The third falter point is women who received ANC, received or purchased, and consumed IFA in any amount. The fourth falter point is women who received ANC and received or purchased recommended ≥180 IFA, and the fifth falter point is women who received ANC and received/purchased and consumed ≥180 IFA.

### 2.4. Explanatory Factors

**Figure 2:**
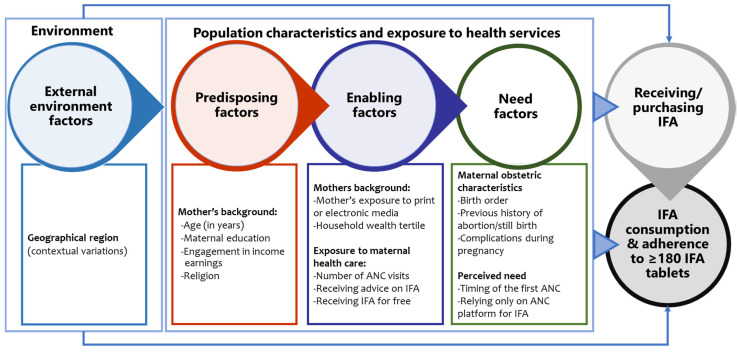
Conceptual diagram of factors influencing receiving and consumption of IFA based on Andersen’s Behavioral Model of health care utilisation and Siekmans et al.’s framework for IFA supplementation [21,33]. ANC—antenatal care, IFA—iron and folic acid.

We used a conceptual framework (Figure 2) to explore the factors associated with receiving and consuming antenatal IFA supplements based on Andersen’s Behavioral Model of health care utilisation and Siekmans et al.’s framework for IFA supplementation [21,33]. A modified version of Andersen’s model underpinned the theoretical framework for explaining health-seeking behaviour, including antenatal health care utilisation in several studies in low-income countries [34,35]. We relied on the versions of the model proposed to explain ANC care-seeking for its relevance to antenatal IFA supplementation [35,36,37]. Our adapted conceptual framework includes three broad domains of factors, predisposing, enabling, and need factors, that influenced the receipt and consumption of antenatal IFA in previous studies from similar settings [23,29,38,39,40,41,42]. We also considered the barriers and enablers of IFA supplementation identified by Siekmans et al., in their conceptual framework [33]. We examined maternal background characteristics such as the mother’s age, education, engagement income earnings, and religion as predisposing factors. Enabling factors consisted of maternal background characteristics such as household wealth and mother’s exposure to print or electronic media, exposure to antenatal healthcare services such as the number of antenatal visits, receiving advice on IFA during pregnancy, receiving IFA free of cost, and relying only on ANC platforms for IFA. We included maternal obstetric characteristics such as birth order, previous history of pregnancy loss and complications during pregnancy, and perceived need for antenatal healthcare, i.e., the timing of the first ANC visit, as the need factors in the conceptual model. We also considered the two districts from the two geographical regions as the external environmental factor.

### 2.5. Statistical Analysis

We used descriptive statistics to analyse and report the participant’s background, obstetric characteristics, and antenatal care-seeking practices. We summarised the number of IFA tablets received, platforms and sources, and the number of IFA tablets consumed during pregnancy by descriptive statistics such as mean (±SD) and proportions for continuous and categorical variables, respectively. We used simple linear regressions to explore the unadjusted associations between the number of IFA received, the number of IFA consumed, and the potential predisposing, enabling, need factors, and external environment indicators included in the conceptual framework (Figure 2).

We checked the linearity assumptions before fitting the linear regressions with continuous independent variables. We added factors associated with the outcomes at a *p*-value < 0.2 in the multiple linear regression models. We tested for interaction between the number of ANC visits and the timing of the first ANC and included an interaction term in the final model. We compared Akaike Information Criteria (AIC) between models with the number of ANC as continuous and categorical variables. We reported mean difference and adjusted mean difference and their 95% confidence interval (CI) from simple and multiple linear regressions, respectively. A *p*-value < 0.05 in the multiple regression models indicated a statistically significant association.

We fitted simple and multiple Poisson regressions for exploring factors associated with user adherence-adjusted coverage of antenatal IFA (consumption of ≥180 tablets) due to its advantage of providing an unbiased estimate of risk ratio (RR) [43]. We reported unadjusted RR and adjusted RR (aRR) for user adherence-adjusted coverage of IFA and their 95% CI from respective Poisson models. Finally, from the multiple Poisson regression, we estimated the expected user adherence-adjusted coverage of IFA in three hypothetical ideal scenarios of universal coverage of programmatically modifiable antenatal healthcare interventions using the “punaf” post-estimation command [44]. Hypothetical scenarios were: (i) all women received four or more ANC visits plus first ANC early (≤4 gestational months in pregnancy), (ii) all women received four or more ANC visits plus first ANC early plus advice on IFA during pregnancy, (iii) all women received eight or more ANC visits plus first ANC early plus advice on IFA during pregnancy. We used Stata (14, StataCorp LLC, College Station, TX, USA) for the analyses and adjusted the multi-stage cluster sampling survey design using the “svyset” command in all analyses [45].

## 3. Results

We interviewed 2572 women who had a live birth six months before the interview. Most mothers (73%) were less than 30 years old and 47% had completed secondary level education (Table 1). Nearly all women (98%) were not in formal employment and about a quarter (26%) had exposure to print or electronic media at least once a week. A little more than a third of the women (37%) were primiparous and 13% had a previous history of pregnancy loss. About a quarter of women received ANC four or more times during pregnancy (26%) and more than one-third (39%) had the first ANC within four months of gestational age.

Overall, 19% of women did not receive or purchase IFA supplements during pregnancy (Table 2). On average, women received or purchased 75 (±62) IFA tablets. Among those who received or purchased IFA supplements in any quantity, 46% received or purchased it only during ANC visits, while 22% from both ANC visits and other sources. The mean (±SD) number of tablets received at each ANC visit was 17 (±15). Of those who received or purchased IFA, approximately equal proportions received IFA supplements from public and private health facilities, 57% and 53%, respectively, while 38% purchased IFA from the pharmacy. On average, women consumed 62 (±58) IFA supplements during pregnancy and one in five women did not consume any IFA supplement.

**Figure 3:**
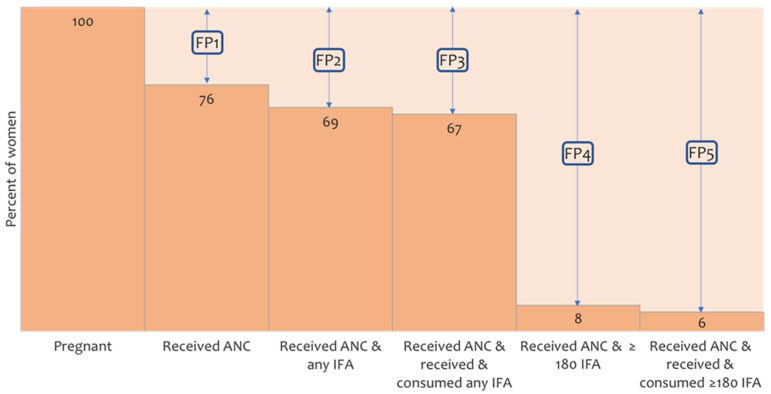
The five falter points (FP) in user adherence-adjusted coverage of IFA supplementation (consumption of ≥180 IFA). FP1—falter in receiving ANC, FP2—falter in receiving any IFA, FP3—falter in consuming any IFA, FP4—falter in receiving adequate IFA (≥180 tablets), FP5—falter in consuming adequate IFA. ANC—antenatal care, IFA—iron and folic acid.

We examined coverage of IFA supplements among women receiving ANC using a falter point analysis. Of all women interviewed, 24% did not receive any ANC during pregnancy (Figure 3). About 69% of women received at least one ANC check-up and IFA supplements in any quantity, resulting in a 7% additional faltering of IFA coverage. A similar proportion (67%) of women received ANC and consumed any IFA. The largest gap (61 percentage points) was between receiving any IFA and adequate (≥180 tablets) IFA supplements. Only 6% of women received ANC and consumed the recommended ≥180 IFA tablets. Consumption of received supplements was higher (*p* < 0.001) if women had received a higher number of supplements, for example, women who received ≤90 IFA tablets consumed 81% of the received tablets, while women who received ≥180 IFA tablets consumed 90% of the received tablets.

Maternal education was associated with the number of IFA supplements consumed during pregnancy (Table 3). On average, women who had higher secondary schooling (≥10 years) and above consumed 24 more supplements (95% CI: 16, 32), and women with secondary education consumed 6 more supplements (95% CI: 1, 11) compared with women who had education up to the primary level. In the multivariable model, women’s age, employment, exposure to mass media, and socio-economic status were not associated with the number of IFA supplements consumed during pregnancy. Obstetric characteristics like birth order of the child and history of previous pregnancy loss also had no association with the number of IFA supplements consumed. However, women who reported pregnancy complications consumed an average of eight tablets (95% CI: −13, −2) less than those who did not have pregnancy complications after adjusting for all other variables in the model. Exposure to maternal health care interventions during pregnancy was strongly associated with antenatal IFA supplement consumption. We found a linear relationship between the number of ANC visits and the number of IFA supplements consumed. This association was modified by the timing of the first ANC visit (*p* < 0.01). Participants who attended ANC services on or before four months of gestational age consumed 14 more IFA tablets (95% CI: 12, 17) for every additional ANC contact. In contrast, women who started ANC on or after five months of gestational age consumed an average of 10 extra IFA tablets (95% CI: 8, 12) for every additional ANC contact. Receiving advice on IFA supplements resulted in women consuming 31 more IFA tablets (95% CI: 24, 38) compared with those who did not receive such advice. Women receiving free IFA supplements had a higher consumption of IFA supplements (14 tablets, 95% CI: 9, 19) than those who did not receive free IFA. However, relying on the ANC service only for IFA supplements resulted in a lower mean consumption of IFA by 39 tablets (95% CI: −45, −33). We found similar associations between background and antenatal care-seeking characteristics and the number of IFA supplements received during pregnancy (Supplement Table S1). However, in contrast to the number of IFA supplements consumed, we did not find any association between maternal pregnancy complications and the number of IFA supplements received during pregnancy.

**Figure 4:**
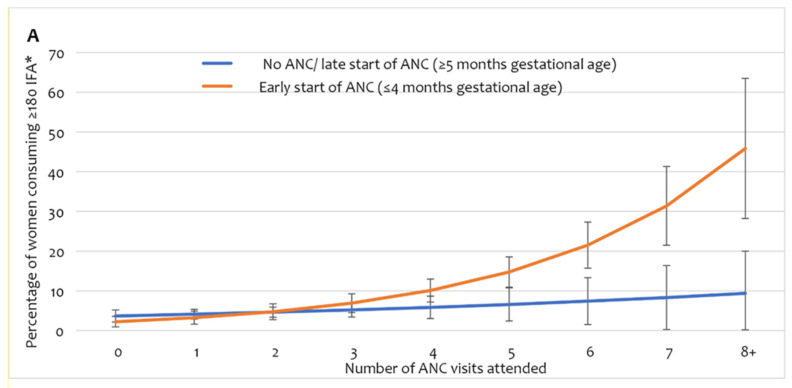

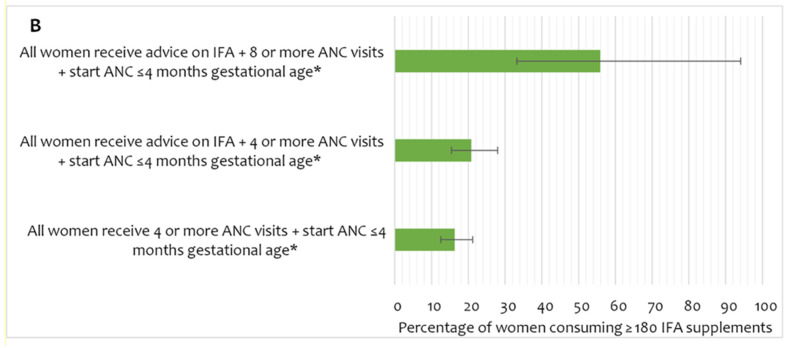
(**A**) User adherence-adjusted effective coverage of IFA supplementation (consuming ≥ 180 IFA) by the number of ANC and timing of first ANC, (**B**) scenario-based projections of user adherence-adjusted coverage of IFA assuming universal coverage of relevant antenatal interventions. * Adjusted for region/area, mother’s education, wealth, exposure to mass media, birth order, receiving free IFA, and receiving IFA only from ANC contracts. IFA—iron and folic acid, ANC—antenatal care.

Adherence to IFA consumption of the recommended dose (user adherence-adjusted coverage of IFA, consumption of ≥180 tablets) showed a stronger interaction effect between the number of ANC visits and the timing of the first ANC received by the women (Figure 4A, Supplement Table S2). The predicted proportion of user adherence-adjusted coverage showed no substantial change in up to three ANC contacts, irrespective of the timing of the first ANC. However, among women who started receiving ANC early, user adherence-adjusted coverage of IFA increased sharply with the number of ANC visits after four or more visits. Among women who began receiving ANC late, user adherence-adjusted coverage of IFA had no considerable increase beyond four or more ANC visits. The scenario-based analysis of expected user adherence-adjusted coverage of IFA assumed a hypothetical universal coverage of antenatal healthcare services. The analysis demonstrates that after adjusting for all other background and obstetric characteristics, if all women received four or more ANC visits and commenced ANC before five months of gestation, the estimated user adherence-adjusted coverage of IFA would be 16% (Figure 4B). If these women received advice on IFA and had four or more ANC contacts, user adherence-adjusted coverage would increase to 21%. Ensuring eight ANC contacts, starting ANC early, and receiving advice on IFA would result in 56% user adherence-adjusted coverage of IFA supplementation in pregnancy.

## 4. Discussion

Adherence to antenatal IFA supplementation is low in Bangladesh [29]. Our study reports the critical predisposing, enabling, and need factors of Andersen’s Behavioral Model of healthcare utilisation that influence receiving and consuming antenatal IFA. We uniquely report the difference in the quantity of IFA supplements received and consumed during pregnancy. The user adherence-adjusted coverage of antenatal IFA supplementation is positively associated with the number of ANC visits, early start of ANC, and receiving advice on IFA. Our modified falter point analysis of IFA coverage fills the data gap on the consumption of adequate ≥180 IFA supplements in the previous analysis [46]. Lastly, our scenario-based projections provide important programmatic decision inputs such as promoting early start and multiple ANC contacts and providing women with advice on IFA at ANC for improving the user adherence-adjusted coverage of antenatal IFA supplementation.

About one in five women did not consume any IFA, which approximates recent estimates from the same geographic regions [25]. We found most women consumed <90 IFA tablets, while previous estimates reported that most women consumed ≥90 tablets [25,29]. The large within-country geographic variations in IFA coverage and the population covered in the previous studies explains this difference [25,47]. Moreover, disruption of antenatal care seeking due to COVID-19 lockdown from late March to May 2020 may have also impacted the total number of IFA supplements received and consumed during pregnancy [48].

Low consumption of adequate IFA tablets is consistent with previous findings from Bangladesh and other low- and middle-income countries [24,29]. Our modified falter point analysis showed that consumption of any IFA was high, and consumption of adequate doses (≥180 tablets) accounted for the largest falter point [24]. Likewise, we did not find an overall difference between receiving and consuming IFA [24]. Our analysis closed the gap in previous research and identified that not receiving or purchasing an adequate number of tablets resulted in low consumption of a sufficient number of IFA supplements. Studies from other South Asian settings, including the supply-side factors of IFA adherence, also indicated that not receiving IFA was a key reason for non-adherence to adequate IFA during pregnancy [21,49]. We found a higher difference between the number of tablets received and consumed among women who received a lower number of tablets (<90). This finding can be explained by the perceived side-effects such as nausea, vomiting, metallic taste, dark stool colour, and constipation that women experience, leading to non-adherence to adequate consumption [41,49,50,51]. We also demonstrated an independent negative association between pregnancy complications and the number of IFA supplements consumed. It is likely that the women who did not face and/or were aware of the side effects of IFA, understood the importance of adequate IFA, received/purchased more tablets repeatedly, and consumed them [21,24].

Consistent with the findings in several previous studies, maternal education was a strong predictor for higher and user adherence-adjusted coverage of IFA supplementation [38,42,52,53]. Women with a higher education are more likely to receive and understand messages on anaemia and IFA, perceive the importance of adequate IFA, understand the health care provider’s advice, read package labelling, and are less affected by the perceived side effects [54]. Educated mothers may also have fewer perceived barriers and misconceptions that are common in the community, for example, consumption of “many” IFA tablets leads to oversized babies with more pregnancy complications and need caesarean section births [22]. Educated mothers are also more likely to have higher autonomy in healthcare decision making, influence over household expenditure, and receive their husbands’ support, which is associated with better adherence to adequate IFA consumption [29,55]. In contrast to previous studies in other low- and middle-income countries, we did not find that a higher maternal household wealth status was associated with receiving and consuming IFA during pregnancy [38,39,42,56]. However, a previous study in Bangladesh reported similar findings [29]. One likely explanation for this is women receive free IFA from public facilities and the cost of purchasing IFA from private sector sources is affordable [21,22,57].

The number of IFA received and consumed and the user adherence-adjusted coverage of IFA were strongly associated with the number of ANC services received. These findings are consistent with the prior evidence [23,24,29,38,42,58]. Our analysis also showed that ANC contacts were the most utilised platform for accessing IFA supplements. Similar to our findings, a study in India also found the timing of the first ANC visit modified the positive association between the number of ANC visits and adherence to adequate IFA consumption during pregnancy [58]. Women who started ANC early are more likely to receive multiple ANC visits and information on IFA, receive or purchase more tablets from multiple visits, and adhere to adequate IFA consumption [54]. In contrast, women who commence ANC late in pregnancy do not have sufficient time to consume ≥180 tablets. Further, the recommendations for starting IFA supplementation in the first trimester in maternal care standard operating procedures and management guidelines for iron deficiency anaemia in Bangladesh are inconsistent [59,60,61].

Women who received IFA only from ANC were less likely to consume an adequate dose. One possible reason is that women are given approximately 20–30 tablets at each ANC visit; they would require multiple visits to receive recommended ≥180 tablets [21,24]. However, only a quarter of the women received four or more ANC visits in our study population. The limited number of IFA tablets provided at each ANC also explains the sharp increase of user adherence-adjusted coverage tablets beyond five or more ANC visits among women who started receiving ANC early. Previous formative research studies in Bangladesh also identified that insufficient and interrupted supply and provision of IFA during ANC at public facilities are important bottlenecks in adequate IFA consumption during pregnancy [21,28]. Several previous studies also support our finding that receiving counselling and information on the importance, recommended number/days, and side effects of IFA during pregnancy influence user adherence to adequate IFA consumption [23,29,40,41,42,54,58]. Receiving counselling or information on IFA may have improved the adherence leading to adequate consumption [21]. This change may have resulted from enhanced awareness of the value of IFA, reduced misconceptions around perceived side-effects, understanding the correct duration of supplementation, knowing alternative sources of IFA, and increasing the frequency of visits to receive IFA.

We acknowledge some limitations of this study. Firstly, we did not have the data on selected supply-side factors, including consistent availably of IFA at the facilities where the women sought ANC and distance to the nearest public or private source of IFA, which we could not include in the multiple regression model. However, our disaggregated data on IFA received from ANC and non-ANC platforms and sources partly address this data gap. We did not ask women about their anaemia status, as routine screening for anaemia is low, and universal antenatal IFA supplementation is recommended [19,62]. Secondly, we did not have observation-based data on the quality of the services provided during ANC at any of the facilities. Thus, we could not analyse the quality-adjusted coverage step proposed in the effective-coverage assessment cascade [26,27]. However, our assessment of user adherence-adjusted coverage stays at a higher endpoint on the effective coverage assessment cascade. Thirdly, the reliance on retrospective self-reported data on the number of IFA tablets received and consumed is prone to response bias. To improve recall, we restricted our participant selection window to six months post-partum. Finally, we can generalise our findings to the rural population as we conducted the study in rural sub-districts. Nonetheless, we propose future research should include an analysis of all steps of the effective coverage cascade following a cohort of women during pregnancy and collecting information on the quality of service at both public and private sources of ANC and IFA and the number of IFA consumed. A detailed qualitative exploration of women’s perception and attitude to IFA and choice of IFA sources would identify the strategies to raise women’s awareness and demand for and compliance to recommend IFA consumption and improve user adherence-adjusted coverage of IFA in pregnancy.

Our study has several policies and programmatic implications for improving user adherence-adjusted coverage of IFA during pregnancy. Emphasis on >4 ANC contacts from health facilities is essential to improve receiving and consumption of antenatal IFA. However, adherence to adequate consumption largely depends on commencing ANC in the first trimester. The inconsistencies in the national ANC protocol and guidelines about starting IFA in the first trimester need correction in line with global guidelines (WHO). Our scenario-based projection suggests that ensuring the current national recommendation of a minimum of four ANC visits would achieve only ~16% user adherence-adjusted coverage [19]. National maternal health and nutrition programmes should consider adopting the 2016 WHO recommendation of eight ANC contacts [11]. A large proportion of women received/purchased IFA from private sector sources. Ensuring quality ANC with counselling on IFA is important in public and private sector ANC contacts. Similarly, increasing the number of supplements provided at each ANC visit, from the current practice of delivering 20–30 IFA tablets, is needed to cover the time until the next appointment. Programmes should explore the targeted community-based distribution of supplements for the women who do not attend ANC multiple times [57].

## 5. Conclusions

This study is the first to report user adherence-adjusted effective coverage of recommended ≥180 IFA tablet consumption during pregnancy in Bangladesh. It identifies the faltering points and factors influencing optimal effective coverage of IFA supplementation in rural areas of Bangladesh. Our findings demonstrate that less than one in ten pregnant women consumed ≥180 IFA tablets during pregnancy. An early start to ANC in the first trimester and a higher number of ANC visits had an incremental benefit in increasing IFA consumption during pregnancy. Interventions targeting the early start of ANC, eight or more ANC contacts, and providing pregnant women with advice on the importance of IFA and IFA supplements in higher quantity at the ANC contacts have the potential to improve effective coverage of IFA during pregnancy.

## Figures and Tables

**Table 1:** Socio-demographic and obstetric characteristics and antenatal care-seeking practices among 2572 women who had a live birth in the six months preceding the interview.

Background Characteristics	Weighted * n	Weighted %
Region		
North (Kurigram)	762	29.6
South (Bhola)	1810	70.4
Mother’s age		
<20	462	18.0
20–29	1409	54.8
30 or more	701	27.2
Mother’s education		
Up to primary	1031	40.1
Secondary	1201	46.7
Higher secondary and above	340	13.2
Religion		
Muslim	2485	96.6
Others	87	3.4
Mother’s employment status		
Employed	65	2.5
Unemployed	2507	97.5
Household Wealth index (tertile)		
Poor	794	30.8
Middle	854	33.2
Rich	925	36.0
Mother’s exposure to print or electronic media (at least once a week)		
No	1905	74.0
Yes	667	26.0
Birth Order of the last child		
1	955	37.1
2	793	30.8
3 or more	825	32.1
History of abortion/stillbirth before this pregnancy		
No	2235	86.9
Yes	337	13.1
Women who had reported complication(s) during pregnancy		
No	1948	75.8
Yes	624	24.2
Number of ANC visits		
No ANC	621	24.2
1–3	1289	50.1
4 or more	662	25.7
Timing of first ANC (N = 1951)		
Early (≤4 months gestational age)	754	38.6
Late (≥5 months gestational age)	1197	61.4

* Weighting adjustment for multi-stage cluster sampling, ANC—antenatal care.

**Table 2:** Women receiving IFA supplements during pregnancy by number of supplements, service delivery platform and source, and consumed IFA supplements.

Indicators	Weighted n	Weighted Mean [±SD] or % (95% CI) ^a^
IFA supplements received		
Mean (±SD) number of IFA supplements received	2572	75 [±62]
Number of IFA supplements by pregnant women		
None	477	18.6 (16.3, 21.1)
1–89	1049	40.8 (38.2, 43.4)
90–179	805	31.3 (28.7, 34.0)
180 or more	242	9.4 (7.9, 11.2)
Platforms for receiving/purchasing IFA supplements	2095	
Only at ANC	956	45.7 (41.9, 49.5)
Only outside ANC	677	32.3 (28.6, 36.3)
Both at ANC and outside ANC	461	22.0 (19.4, 24.9)
Mean (±SD) IFA received at each ANC contact	1958	17 [±15]
Source of IFA supplements (multiple responses)	2095	
Home	428	20.4 (18.0, 23.1)
Public health facilities	1188	56.7 (53.4, 60.0)
Private hospital/doctor	1107	52.8 (49.0, 56.6)
Pharmacy/drug shop	796	38.0 (34.5, 41.6)
NGO health facility	63	3.0 (2.2, 4.2)
Others	43	2.0 (1.4, 3.0)
IFA supplements consumed		
Mean (±SD) number of IFA supplements consumed	2572	62 [±58]
Number of IFA supplements consumed		
None	527	20.5 (18.2, 23.0)
1–89	1205	46.8 (44.2, 49.5)
90–179	663	25.8 (23.2, 28.6)
180 or more	178	6.9 (5.8, 8.3)

SD—standard deviation, IFA—iron and folic acid, ANC—antenatal care, NGO—Non-Government Organisation; ^a^ weighted % if not mentioned otherwise in the row.

**Table 3:** Factors associated with the number of IFA supplements consumed.

Variables	N (Weighted)	Number of IFA Consumed		
	2572	Mean (±SD)	Mean Difference (95% CI)	Adjusted Mean Difference (95% CI)
Region/area				
North (Kurigram)	762	71.7 (±82.1)	Ref	Ref
South (Bhola)	1810	58.1 (±46.2)	−13.5 (−20.9, −6.2)	2.2 (−3.2, 7.6)
Mother’s age				
<20	462	64.2 (±56.3)	Ref	Ref
20–29	1409	65.4 (±57.3)	1.3 (−5.5, 8.0)	6.0 (−0.0, 12.1)
30 or more	701	54.1 (±58.6)	−10.0 (−17.3, −2.8)	3.4 (−4.04, 11.9)
Mother’s education				
Up to primary	1031	46.7 (±50.6)	Ref	Ref
Secondary	1201	65.7 (±57.3)	19.0 (13.9, 24.1)	6.0 (1.2, 10.8)
Higher secondary and above	340	96.2 (±62.2)	49.4 (39.5, 59.3)	23.6 (15.8, 31.5)
Religion				
Muslim	2485	61.5 (±57.5)	Ref	Ref
Other	87	80.8 (±59.7)	19.3 (5.3, 33.2)	9.2 (−1.8, 20.1)
Work involvement				
Not employed	2507	61.8 (±57.4)	Ref	Ref
Employed	65	74.9 (±69.9)	13.1 (−4.0, 30.3)	6.5 (−8.3, 21.3)
Household Wealth (tertile)				
Poor	794	53.5 (±56.8)	Ref	Ref
Middle	854	56.6 (±54.9)	3.1 (−4.1, 10.2)	1.5 (−4.2, 7.2)
Rich	925	74.6 (±58.5)	21.1 (13.9, 28.3)	3.0 (−3.4, 9.4)
Mother’s exposure to print or electronic media (at least once a week)				
No	1905	58.0 (±54.1)	Ref	Ref
Yes	667	74.1 (±66.2)	16.1 (9.5, 22.7)	4.2 (−1.5, 9.9)
Birth Order of the last child				
1	955	72.3 (±57.7)	Ref	Ref
2	793	62.7 (±60.2)	−9.6 (−16.5, −2.7)	−4.5 (−10.0, 1.1)
3+	825	49.8 (±52.9)	−22.4 (−29.1, −15.7)	−5.2 (−12.7, 2.3)
Any history of abortion/stillbirth before this pregnancy				
No	2235	61.8 (±57.3)	Ref	**
Yes	337	64.3 (±60.6)	2.5 (−5.2, 10.3)	**
Women who had pregnancy complications				
No	1948	70.8 (±58.9)	Ref	Ref
Yes	624	66.2 (±53.8)	5.4 (−2.0, 12.7)	−7.8 (−13.0, −2.0)
Number of ANC and timing of first ANC				
Number of ANC visits among mothers who received none or started late (≥5 months GA) ^α^	1818	19.2 (±28.5) ^α^	11.2 (9.4, 13.0)	9.8 (7.8, 11.8)
Number of ANC visits among mothers who started early (≤4 months GA) ^α^	754	25.7 (±20.3) ^α^	14.4 (11.7, 17.1)	14.1 (11.5, 16.6)
Received advice on IFA				
No	1232	42.9 (±52.2)	Ref	Ref
Yes	1340	79.8 (±56.9)	36.9 (30.9, 43.0)	30.9 (24.2, 37.5)
Received IFA free				
No	1498	50.5 (±55.6)	Ref	Ref
Yes	1074	78.4(±56.5)	27.9 (22.2, 33.5)	14.1 (9.3, 18.8)
Received IFA only from ANC contacts				
No	1616	60.2 (±59.4)	Ref	Ref
Yes	956	65.4 (±53.1)	5.2 (−0.4, 10.8)	−39.2 (−45.0, −33.2)

SD—standard deviation, IFA—iron and folic acid, ANC—antenatal care, GA—gestational age; ^α^ Mean (±SD) refers to the mean number of IFA consumed per ANC visit among women receiving the first ANC late (≥5 months of gestational age) and early (≤4 months of gestational age), ** variable not included in the adjusted model.

## Data Availability

Data supporting reported results can be found from https://figshare.com/articles/dataset/Coverage_of_IFA_supplementation_in_pregnancy/20105399 (accessed on 29 June 2022).

## Supplementary Materials

The following supporting information can be downloaded at: https://www.mdpi.com/article/10.3390/nu14153114/s1, Table S1: factors associated with the number of IFA supplements received; Table S2: factors associated with user adherence-adjusted effective coverage (consumed 180+ tablets) during pregnancy.
