# Supplementary material for: Iron and Folic Acid Supplementation in Pregnancy: Findings from the Baseline Assessment of a Maternal Nutrition Service Programme in Bangladesh
Source: Nutrients. 2022 Jul 28;14(15):3114. doi: 10.3390/nu14153114 (PMC9370216; doi:10.3390/nu14153114)
Supplement: Supplementary file 1 [file nutrients-14-03114-s001.zip › nutrients-1819231-supplementary.pdf]

Table S1: Factor associated with the number of IFA supplements received

Variables	N(weighted)	Number of IFA received	
	2572	Mean difference (95% CI)	Adjusted mean difference (95% CI)
**Region/area**			
North (kurigram)	762	Ref	Ref
South (Bhola)	1810	-11.7(-20.1, -3.9)	7.3(1.5, 13.0)
**Mother's age**			
<20	462	Ref	Ref
20-29	1409	-3.3(-11.5, 5.0)	1.8(-5.1, 8.6)
30 or more	701	-16.7(-25.3, -8.1)	-1.5(-9.5, 6.6)
**Mother's education**			
Up to primary	1031	Ref	Ref
Secondary	1201	20.1(14.2, 26.1)	4.1(-1.3, 9.6)
Higher secondary and above	340	54.5(44.3, 64.7)	25.6(17.3, 33.9)
**Religion**			
Muslim	2485	Ref	Ref
Others	87	18.4(2.0, 34.8)	7.9(-5.8, 21.6)
**Work involvement**			
Not employed	2507	Ref	Ref
Employed	65	12.0(-5.3, 29.4)	7.2(-7.3, 21.7)
**Household Wealth (tertile)**			
Poor	794	Ref	Ref
Middle	854	3.9(-3.9, 11.7)	1.9(-4.2, 8.1)
Rich	925	23.8(16.0-31.5)	3.6(-3.6, 10.8)
**Mother's exposure to print or electronic media (at least once a week)**			
No	1905	Ref	Ref
Yes	667	17.4(10.4, 24.5)	5.1(-0.9, 11.1)
**Birth order of the last child**			
1	955	Ref	Ref
2	793	-13.9(-21.5, -6.2)	-5.8(-12.0, 0.3)
≥3	825	-27.7(-34.7, -20.6)	-5.6(-13.1, 1.8)
**Any history of abortion/stillbirth before this pregnancy**			
No	2235	Ref	*
Yes	337	2.3 (-6.3, 10.8)	*
**Women who had pregnancy complications**			
No	1948	Ref	Ref
Yes	624	9.3(0.9, 17.7)	-5.5(-11.0, 0.3)

Variables	N(weighted)	Number of IFA received	
	**2572**	**Mean difference (95% CI)**	**Adjusted mean difference (95% CI)**
**Number of ANC and timing of first ANC**			
Number of ANC visits among mothers who received none/ started late (≥5 months GA) α	1818	13.4(11.6, 15.2)	11.1(9.1, 13.2)
Number of ANC visits among mothers who started early (≤4 months GA) α	754	15.8(12.8, 18.8)	15.3(12.6, 18.0)
**Received advice on IFA**			
No	1232	Ref	Ref
Yes	1340	44.2(37.8, 50.7)	31.8(24.6, 39.0)
**Received IFA free**			
No	1498	Ref	Ref
Yes	1074	36.6(30.7, 42.5)	21.2(16.2, 26.2)
**Received IFA only from ANC contacts**			
No	1616	Ref	Ref
Yes	956	11.3(5.6, 16.9)	-37.6(-43.5, -31.6)

IFA – iron and folic acid, ANC – antenatal care, GA – gestational age, * variables not included in the adjusted model

Table S2: Factor associated with user adherence-adjusted effective coverage (consumed 180+ tablets) during pregnancy

Variables	N(weighted)	Women consumed 180+ tablets	
	2572	Risk Ratio	Adjusted Risk Ratio
**Region/area**			
North (kurigram)	762	Ref	Ref
South (Bhola)	1810	0.49(0.35-0.69)	0.65(0.48-0.88)
**Mother's age**			
<20	462	Ref	
20-29	1409	1.34(0.83-2.17)	*
30 or more	701	1.00(0.60-1.65)	*
**Mother's education**			
Up to primary	1031	Ref	Ref
Secondary	1201	1.67(1.15-2.44)	1.21(0.80-1.81)
Higher secondary and above	340	4.21(2.74-6.48)	2.2(1.29-3.82)
**Religion**			
Muslim	2485	Ref	*
Others	87	1.39(0.68-2.85)	*
**Work involvement**			
Not employed	2507	Ref	*
Employed	65	1.54(0.74-3.19)	*
**Household Wealth (tertile)**			
Poor	794	Ref	Ref
Middle	854	0.94(0.58-1.51)	0.90(0.58-1.41)
Rich	925	1.76(1.22-2.56)	0.97(0.63-1.51)
**Mother's exposure to print or electronic media (at least once a week)**			
No	1905	Ref	Ref
yes	667	1.84(1.38-2.45)	1.21(0.88-1.65)
**Birth order of the last child**			
1	955	Ref	Ref
2	793	1.03(0.73-1.45)	1.09(0.80-1.48)
≥3	825	0.55(0.34-0.73)	0.89(0.56-1.41)
**Any history of abortion/stillbirth before this pregnancy**			
No	2235	Ref	*
Yes	337	1.10 (0.69-1.77)	*
**Women who had pregnancy complications**			
No	1948	Ref	*
Yes	624	0.82(0.54-1.24)	*
**Number of ANC and timing of first ANC**			

Variables	N(weighted)	Women consumed 180+ tablets	
	2572	Risk Ratio	Adjusted Risk Ratio
Number of ANC visits among mothers who received none or started late (≥5 months GA)	1818	1.11(0.94, 1.31)	1.24(0.94, 1.34)
Number of ANC visits among mothers who started early (≤4 months GA)	754	1.49(1.33, 1.65)	1.46(1.32, 1.62)
**Received advice on IFA**			
No	1232	Ref	Ref
Yes	1340	1.68(1.18-2.38)	1.52(1.03, 2.23)
**Received IFA free**			
No	1498	Ref	Ref
Yes	1074	1.29(0.92-1.81)	0.95(0.66, 1.36)
**Received IFA only from ANC contacts**			
No	1616	Ref	Ref
Yes	956	0.66(0.45-0.97)	0.30(0.20, 0.70)

IFA – iron and folic acid, ANC – antenatal care, GA – gestational age, * variables not included in the adjusted model.
